# Functional clustering of yeast proteins from the protein-protein interaction network

**DOI:** 10.1186/1471-2105-7-355

**Published:** 2006-07-24

**Authors:** Taner Z Sen, Andrzej Kloczkowski, Robert L Jernigan

**Affiliations:** 1 L.H. Baker Center for Bioinformatics and Biological Statistics, Iowa State University Ames, IA 50011, USA; 2 Department of Biochemistry, Biophysics, and Molecular Biology, Iowa State University, Ames, IA 50011, USA

## Abstract

**Background:**

The abundant data available for protein interaction networks have not yet been fully understood. New types of analyses are needed to reveal organizational principles of these networks to investigate the details of functional and regulatory clusters of proteins.

**Results:**

In the present work, individual clusters identified by an eigenmode analysis of the connectivity matrix of the protein-protein interaction network in yeast are investigated for possible functional relationships among the members of the cluster. With our functional clustering we have successfully predicted several new protein-protein interactions that indeed have been reported recently.

**Conclusion:**

Eigenmode analysis of the entire connectivity matrix yields both a global and a detailed view of the network. We have shown that the eigenmode clustering not only is guided by the number of proteins with which each protein interacts, but also leads to functional clustering that can be applied to predict new protein interactions.

## Background

Systems biology is a new frontier for bioinformatics research, aimed at understanding complex biological systems in cells by integrating interactions between large numbers of constituent components, including genes, proteins, and metabolites. Examples of systems biology research include studies of gene interaction networks [1-3], regulatory networks[1,4-6], metabolic pathway modeling[7,8], and combinations of these networks[3,9-11]. By its nature, systems biology studies require highly detailed, large-scale simulations that are computationally demanding.

Proteins represent the major category of large functional biomolecules. How proteins interact with one another is a current subject of many high-throughput studies. The number of proteins in an organism can reach tens of thousands. Comprehending the functional, developmental, and regulatory networks comprising these temporal and spatial protein pairs is a formidable task [12-17] since the number of their pairwise combinations can reach millions.

Protein clustering in global interaction networks is important for revealing cellular functionality (for example [18]). Clustering usually involves defining one or more properties among samples and forming individual clusters based on the similarities of these properties, such as association with similar biochemical pathways (e.g. metabolic, signaling, regulatory), functional classification, cellular localization, or evolution (co-evolution, conservation, phylogeny). Usually, though, a combination of properties have been utilized [19,20]. Clustering in part serves to detect incorrect annotations in databases (e.g. GO or KEGG), or to discover new connections in interaction networks. Clusters based on the topological information[21,22] itself can also be useful to understand the organizational principles of interaction networks (not only biological, but also social networks[23]) and to identify highly interconnected proteins with functional significance[24].

Our approach in this paper using connectivity matrix and subsequent eigenvalue/eigenvector decomposition is also based on the topological properties of the interaction network as a whole. Although significant proteins (in each eigenvector) form clusters, these clusters differ from those obtained by methods that are based solely on protein properties, because they reflect the organizational patterns of the protein interactions themselves based on topological considerations.

In the present study, we show that computational analyses of experimental data on protein networks can lead to discoveries of new, unexpected relationships, which emphasizes the importance of the global view of a protein interaction network.

## Results and discussion

In this paper, we have used spectral analysis of graphs methodology. We earlier applied a similar approach for protein dynamics analysis using elastic network models [25-29]. Spectral analysis has also been applied by Vishveshwara and co-workers to the problems of protein structure similarity, protein domain identification, and backbone clustering [30-35]. They have shown that important clusters in protein structures can be extracted from dominant eigenvalues of Kirchhoff's matrix [33], and that the vector components of the second lowest eigenvalue of the Kirchhoff's matrix of protein-structure similarity network leads to successful sub-clustering of functionally similar proteins [32]. Biological networks were also extensively studied by Alon, Leibler and co-workers [36-50].

We used the yeast protein interaction data available in the GRID (General Repository for Interaction Data sets)[51] database, which is a curated database of physical, genetic, and functional interactions encompassing many data sets [52-58]. The database contained 4906 proteins, and 19,037 interactions at the time we start our analysis. Most recently, this database has been updated by the addition of 753 new experimental interactions determined by Krogan *et al*. [59]. Although the whole set of interactions is not curated and therefore includes some redundancies with previous entries in the database, it nevertheless reveal significant new interactions. This has created an excellent opportunity for us to test the predictive power of our method. Our preliminary studies based on the previous version of the database (not containing Krogan *et al*.'s results) show that our theoretical approach leads to correct predictions of some new protein-protein interactions provided in Krogan *et al*.'s data. Details of some of these successfully predicted cases will be shown here.

### Connectivity matrix and its eigenmode analysis

We have converted the pairwise interaction information obtained from the GRID data into a connectivity matrix **C **for subsequent analyses. The individual elements of the symmetric matrix **C **are as follows: 1, if two proteins interact; 0, if they do not; and the diagonal elements of the contact matrix are taken as the negative sum of the other row (or column) elements. We then applied the standard method of matrix eigenanalysis used in algebra. Readers who are non-experts in this field may read a brief tutorial provided in the Methods section.

We should note that our definition of the diagonal elements of the connectivity matrix as sums of all non-diagonal elements of the given column (or row), automatically leads to a connectivity matrix that is singular, and must be analyzed through the Singular Value Decomposition technique. The definition of diagonal elements of the matrix that implies its singularity has some deep physical meaning when the technique is applied to protein structures. For example in the case of elastic network models of proteins [25-29], or Gaussian model of polymer networks [60,61], the zero eigenvalues are associated with the motion of the center of mass of the studied object.

**Figure 1:**
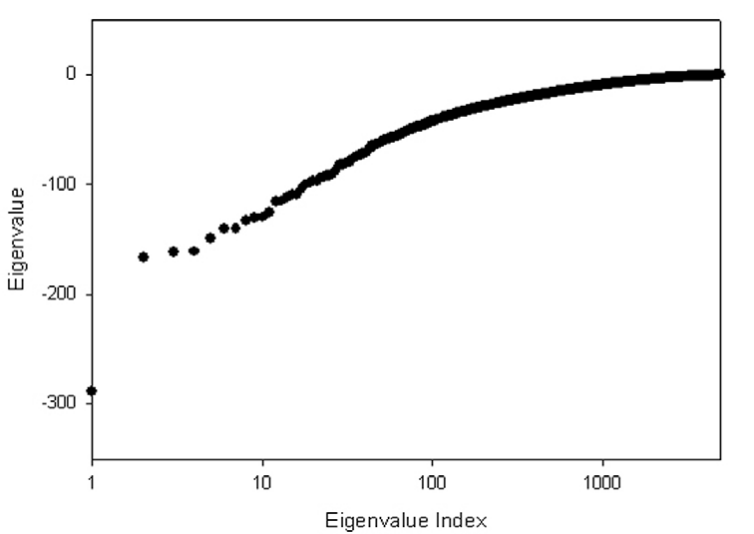
Eigenvalue distribution for the yeast protein-protein interaction network.

A similar study of the protein-protein interaction network in budding yeast by using spectral graph theory has been published by Bu *et al*. [62]. Their approach follows the Gibson, Kleinberg and Raghavan's analysis of Internet Web topology [63]. There is however a significant difference between our method and theirs, because of the use of different matrices and completely different methodology of clustering. The connectivity matrix used by Bu et al. has the diagonal elements defined as zeros. Such definition of diagonal elements of the matrix leads to both positive and negative eigenvalues. In our case, all eigenvalues are always negative as seen from Fig. 1. Additionally Bu *et al*. [62] were looking for quasi-cliques and quasi-bipartites by applying the Gibson, Kleinberg and Raghavan [63] iteration method, and by using the following criteria: (i) 10% of top eigenvectors were selected, (ii) each protein had to interact with 20% of members, and (iii) the minimal size of quasi-cliques was set to 10. The resulting 48 clusters had sizes between 10 and 109 and were characterized by significant functional similarity. Our approach is fundamentally different, since in our case, the number of clusters is much greater (4906) and a given protein can belong to many eigenclusters. The striking similarity between our results and Bu et al. is, however, that each eigencluster is characterized by functional similarity of member proteins.

#### Singular Value Decomposition (SVD) computations

In this work, we used SVD (since det **C **= 0) to extract eigenvalues and eigenvectors instead of alternative clustering methods. The SVD method has been extremely useful in the development of elastic network models[25,26] used to compute protein motions (i.e. to divide the structure into domains(structure clusters) and in its applications[64], microarrays[65], as well as for analysis of other complex data[66]. The SVD methodology has been recently used to study network-level analysis of metabolic regulation in the human red blood cells [67], detection of functional connectivities in cortical thickness [68], studies of transcription modules in large-scale gene expression data [69], or analysis of large-scale metabolomic networks [70,71].

We have applied the SVD subroutine available in the LAPACK[72] library to calculate all eigenvalues and eigenvectors of the connectivity matrix. This is a straightforward procedure and requires a relatively modest expenditure of computing time: All of 4906 eigenvalues (and corresponding eigenvectors) has been computed in 3 hours on a SGI Origin 2800 with 6GB of RAM. We have found that all eigenvalues are negative except for 43 zero eigenvalues. This behavior showing a relatively large numbers of zero eigenvalues typically implies a network with some sparsely connected nodes, as we have previously observed in our analysis of protein structures[25].

**Figure 2:**
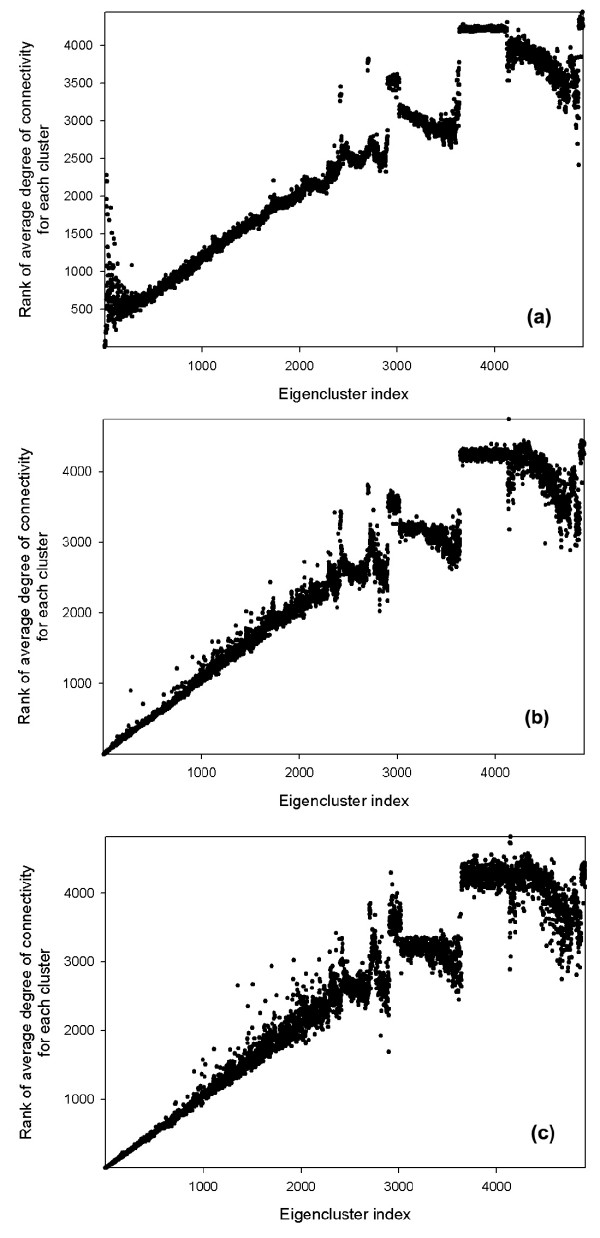
**The rank of the average degree of connectivity of eigenclusters as a function of the eigenvector index **for the cutoff values of (a) 0.01, (b) 0.05, (c) 0.1

The sorted eigenvalues of the connectivity matrix for the yeast protein-protein interaction network are shown in Figure 1. We have used the semi-logarithmic scale for the plot, where only the abscissa (x-axis) of the plot scales logarithmically, for additional emphasis on the shape of eigenvalue distribution. The eigenvalue index in Fig. 1 refers to the position in ranking of eigenvalues. After ranking the eigenvalues, we analyzed the corresponding eigenvectors in a more detailed way. We observed that the number of significant components in a given eigenvector is always quite small compared to the total number of components. We defined an *ad hoc *cutoff value for the systematic classification of these significant components. After testing different cutoff values, we treated an eigenvector component as significant if its absolute value was above 0.05. Fig. 2 shows the plots of the rank of the average degree of connectivity of eigenclusters as a function of the eigenvector index for three cutoff values (a) 0.01, (b) 0.05, and (c) 0.1. The eigenvector indices above 2500 correspond to significant dispersion of the data; therefore, we will compare the plots for the eigenvectors approximately up to 2500. In the case of the 0.01 threshold (Fig. 2a) the left-hand of the plot is very noisy. An increase of threshold to 0.1 (Fig. 2c) diminishes the noise at the left-hand of the plot, but unfortunately, it increases noise for the eigenvectors in the range of 1000–2500. The rationale of choosing 0.05 (Fig. 2b) as the threshold value, therefore, is to reduce the noise not only for the left-hand of the plot, but also for the rest of the plot. Also, the linear relationship of the average degree in the eigenclusters in Figure 2b in part justifies the choice of 0.05 as a threshold.

**Figure 3:**
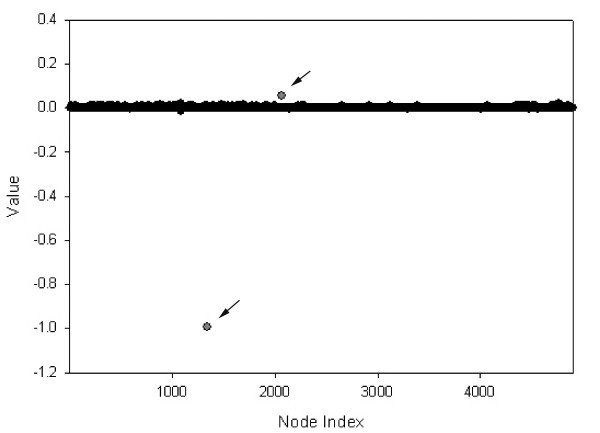
**Significant proteins corresponding to eigencluster #21**. Only two proteins represent significant non-zero components: SER3 (YER081W) with a value of -0.99, and SLT2 (YHR030C) with a value of 0.055 (shown with arrows). Interestingly, both of these proteins have 96 neighbors.

As an example showing how we have chosen significant proteins in an eigenvector, Fig. 3 shows eigenvector #21. In this eigenvector, there are only two points (indicated by arrows) corresponding to the most significant components. Note that the *i*^th ^component of the eigenvector corresponds to the *i*^th ^protein in the connectivity matrix **C**. In this way, we may define an eigencluster as a set of proteins corresponding to the most significant components of a given eigenvector.

We should note that the same protein(s) may belong to several different clusters. This is because each cluster corresponds to an eigenvector related to a specific eigenvalue. Because the whole protein interaction network database for yeast contains 4906 proteins, there are also 4906 eigenvectors, and corresponding eigenclusters. Since each cluster contains at least several proteins, every protein belongs usually to several clusters. This corresponds to the situation in the normal mode analysis of protein motions, where a given residue can be involved at once in several functionally important motions, which may lead to functional promiscuity of proteins[73], where the same protein can have several different functions.

### The inherent limitations in interaction maps

Interactome datasets contain protein interaction information obtained using a wide range of experimental methods, each providing data with differing reliability due to the limitations of the method used. One approach to reconcile the reliability of the protein interaction data obtained using various experimental methods is to assign weights to the interactions based on either the confidence of the particular experimental method, or the confirmation of interactions by additional experimental methods (e.g. ref [19,74]). However, this approach also creates additional problems: not every protein interaction can be verified with multiple-experiments due to experimental limitations, and such weighting schemes might increase the unreliability of the data.

Classical wet-bench molecular biology approaches that focus on a single protein interaction are generally accurate. However, when a high-throughput method (e.g. the yeast two-hybrid assays) is used, the number of wrongly annotated interactions (i.e. false positives) increases, and sometimes, even some reported protein interactions cannot be reconciled with the known protein complexes[75].

The exact false positive rates and completeness of these large-scale experiments are relatively unknown because of coverage limitations: When Vidal and co-workers[76] created random, exponential, power-law, and truncated normal topologies, they observed that these sampled maps were not characteristically different from those obtained using the yeast two-hybrid systems, suggesting that the current interaction maps may be much less complete than we previously thought. The incompleteness of the protein networks thus biases topology-based analyses[77].

Another limitation in protein networks is that global protein interaction networks present a rather static picture of protein interactions, neglecting transport and kinetic aspects. There are two distinct *in vivo *requirements for proteins to interact: first, two proteins need be in close proximity inside the cell; second, the kinetics of this interaction depends on their concentration and diffusion limitations. These limitations are coupled with other cellular processes regulating gene expression and utilizing embedded positive and negative control loops to ensure cell fitness. In the analysis of protein interaction networks, these issues are usually overlooked for practical reasons.

Despite these difficulties, computational analyses of protein interaction networks could be extremely useful, for example, if they can suggest new likely pairings that have not been yet discovered, or reveal new structural or functional linkages within clusters of proteins from the protein network.

### The yeast GRID database: physical, genetic, and functional interactions

The GRID database used for our calculations contains not only physical interactions, but also genetic and functional interactions. We should keep in mind that the available physical interactions in the database cannot always be understood in the sense that two molecules are selectively and specifically binding *in vivo*. For example, even the results of the yeast two-hybrid experiments that use hunt and bait plasmids may suffer from the presence of promiscuous hydrophobic patches on a protein surface, so an experimentally derived interaction may not correspond with certainty to an actual *in vivo *interaction. The definitions for genetic and functional interactions are even less strict: the interaction data obtained with the synthetic lethality experiments might instead indicate that two "interacting" proteins were only in closely related pathways/processes.

### The Protein contact matrix decomposition leads to clusters of proteins having similar numbers of interactions

The rank of the average degree of connectivity for eigenvector clusters is shown in Figure 2 as a function of the eigenvector index. The rank grows almost linearly up to approximately the 2200^th ^cluster, which is an interesting finding in itself. The remainder of the clusters contains proteins with small numbers of neighbors, and as a result the presence of noise disturbs the linearity of the plot. Figure 2 reveals that the singular value decomposition method clusters proteins according to their numbers of interactions (degree). In the spectral theory it is known that eigenvectors will cluster nodes with similar degrees of connectivity. The most crucial discovery in our work is that nodes that have similar degree of connectivity are highly likely to interact with each other. This observation might be possibly used in searches for missing protein-protein interactions.

### Highly non-random nature of interaction clustering

**Figure 4:**
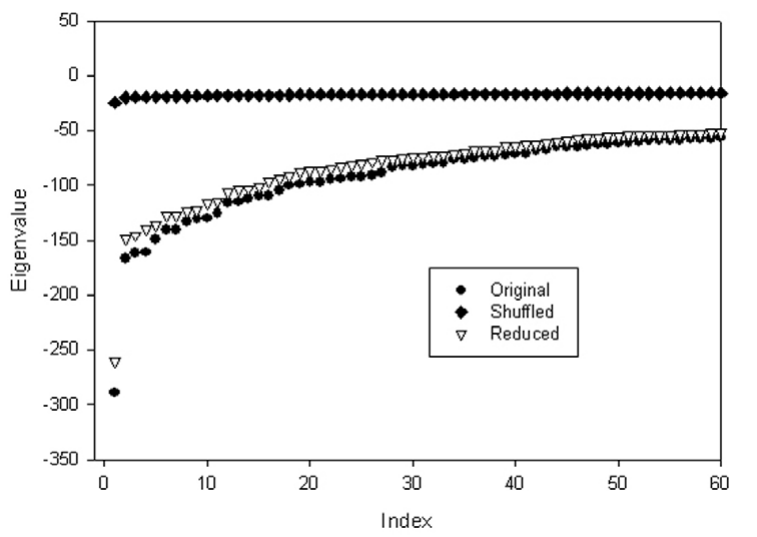
**Comparison of eigenvalue distributions **for (a) the GRID yeast protein interaction matrix (denoted as Original), (b) the randomly shuffled matrix that has same number of connections as the original matrix (Shuffled), and (c) the matrix that has 10% fewer interactions than the original matrix (Reduced). There is a clear difference between randomly shuffled case and the original or reduced GRID data sets.

To investigate whether the observed clustering of interactions detected by the eigenanalysis of the GRID database is random, we have performed a simple numerical experiment. We have compared the distribution of eigenvalues for three different cases: for the original GRID yeast dataset matrix; for the same matrix but with randomly shuffled interactions; and for the matrix obtained from the original one by randomly removing 10% of interactions. The results shown in Fig. 4 clearly demonstrate that the GRID yeast interaction matrix contains a high degree of non-randomness. The comparison between the original and the shuffled matrices proves that SVD clustering has a significantly non-random character. Note that the yeast protein network, similar to other scale-free networks[78], presents unusual properties when protein pairs are randomized. For example, in a recent study, Maslov and Sneppen[79] kept the degree for each protein constant, and randomly shuffled the interacting pairs. After this partial shuffling, they observed that the connections between highly and low-connected pairs were more conserved than the connections between highly connected pairs. Here, however, we apply full shuffling: random assignments of both degrees and interacting pairs to understand the dependence of eigenvalue distribution on the topology of the interaction map.

The clusters revealed by the eigenanalysis show order and contain functional information. This is an important observation motivating further, more detailed studies. The distributions of eigenvalues of the original and the reduced matrices, in contrast to the shuffled matrices, are quite similar. This proves that despite possible experimental errors and many undiscovered interactions in the GRID database, the overall shape of the eigenvalue distribution and the resulting interaction clusters are conserved. This conservation can be exploited for predictive purposes.

### Eigenvector cutoff and node connectivities

We have investigated all eigenmodes in our analysis. For each eigenvector, we have tabulated all the proteins corresponding to components having absolute values above 0.05 and have examined the connectivities among them as specified by the GRID database. We have also calculated the number of neighbors (degree of connectivity) for each protein. We found that the protein with the highest number of protein connections is JSN1 (YJR091C – names in parentheses are the systematic names, called sometimes ORF names/numbers) with 288 neighbors. The protein that has the second highest number of neighbors is YKE2 (YLR200W), with 166 neighbors. In our analysis, JSN1 is the protein that corresponds to the smallest eigenvalue, and YKE2 corresponds to the second smallest eigenvalue (Table 1). For some eigenvalues there are multiple proteins corresponding to components of the eigenvector with absolute values larger than the cutoff, but nonetheless the number of neighbors found for proteins within a cluster varies within a rather narrow range in comparison with the size of the whole protein interaction network.

A critical question remains: are these clusters formed solely according to the number of interacting proteins (i.e. spectral clustering)? Or does the function of proteins influence clustering (i.e. functional clustering)? The data we provide in this paper support the functional clustering hypothesis.

The spectral and functional nature of clusters may not be exclusive: their detailed nature could drive evolution in such a manner that the function of the protein is influenced not only by its functional type, but also by the number of protein neighbors in the whole network in order to create some vital control mechanisms to support cellular fitness. We will explore the presence of functional clustering in the following examples.

### Extracting sub-nets with significant interconnections (Eigenvector #23)

**Figure 5:**
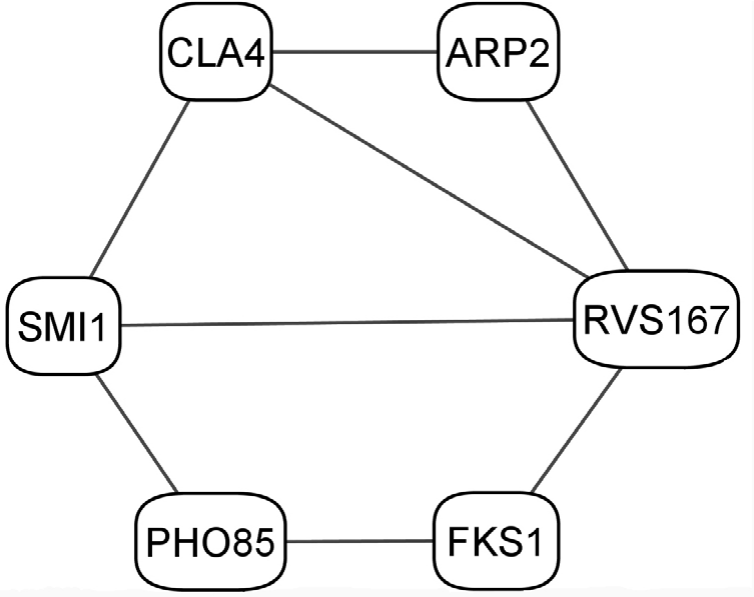
**Connections for the proteins interacting within cluster #23**. The edges represent experimental protein interactions.

In the case of eigenvector #23, there are 6 significant proteins. These proteins, shown in Table 2 and Figure 5, form a functionally related network. For example, ARP2 (YDL029W), RVS167 (YDR388W), FKS1 (YLR342W), CLA4 (YNL298W) are actin-related proteins, whereas PHO85 (YPL031C) and SMI1 (YGR229C) are involved with the cell-cycle. Although the annotations given for these two sets of proteins seem to differ, there is experimental evidence of interactions as shown in Figure 5 implying some functional relationship. There could also be a functional interaction between proteins ARP2 (YDL029W) and FKS1 (YLR342W), not captured by the GRID database. The function of ARP2 is annotated in the GO database as "actin binding" during the process of actin filament organization. On the other hand, FKS1 has 1,3-beta-glucan synthase activity and is a part of the actin cap in *S.cerevisiae*[80]. These annotations merely suggest some interaction via actin; however, further experimentation is necessary to firmly establish whether a physical interaction can occur between these two proteins.

### Sub-nets with few interconnections – are there missing links? (eigenvector #67)

**Figure 6:**
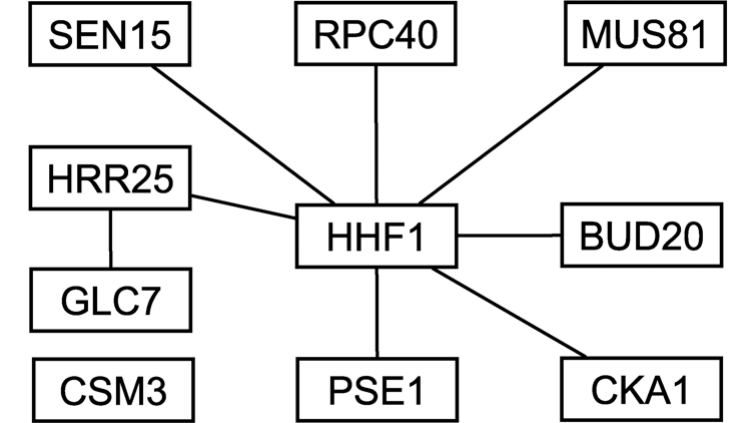
**The protein cluster from eigenvector #67, which has a star-like form**. This form offers a major contrast with that of the cluster shown in Fig. 5, which has more interconnections. CSM3 (YMR048W) is unconnected to the rest of the proteins in this cluster.

The cluster for eigenvector #67 has more proteins than do the clusters for eigenvectors #21 and #23. The significant proteins in this eigencluster are shown in Table 3 and Figure 6. The numbers of neighbors for each protein in this cluster lie within the range of 42 and 67. Interestingly, all the significant proteins corresponding to eigenvector #67 form a cluster with an imperfect star geometry. Note that decreasing the cutoff value will increase the number of proteins belonging to the eigencluster, but these new proteins may or may not interact with other proteins.

There is also a question as to whether CSM3 may in fact be functionally disconnected from the other proteins in this cluster as suggested in the GRID database. Is it possible to functionally relate CSM3 to other proteins in the cluster? The function of CSM3 is currently unknown according to the GRID database, however, it is known that the protein participates in meiotic chromosome segregation and DNA replication[81]. We cannot reach a definite conclusion as to whether CSM3 interacts with any other member of the cluster; the confirmation of these putative interactions must rely on future experimental studies, but the present analysis may be useful in suggesting this specific possibility out of the millions of others.

### Even small subnets that are not fully connected may have missing links (eigenvector #4850)

**Figure 7:**
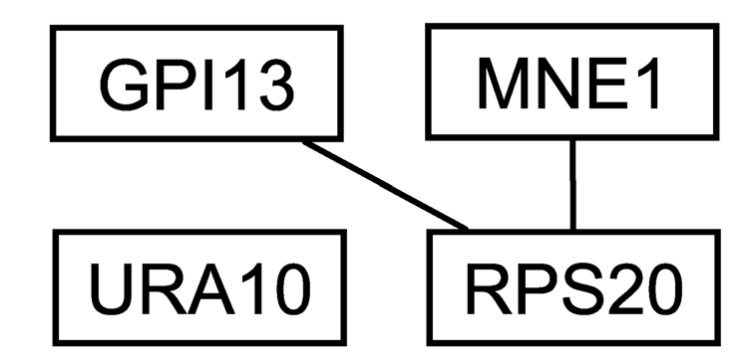
**The three proteins connected in cluster #4850**. The fourth protein URA10 (YMR271C) present in this cluster is not connected to the three shown here.

For the upper end of eigenvalue distribution, we have analyzed the case for eigenvector #4850. The connectivities and GO annotations for this cluster are also shown in Fig. 7. Four significant proteins in this cluster are shown in Table 4: GPI13, RPS20, URA10, and MNE1. Experimental evidence given in the GRID database substantiates the interaction between RPS20 and GPI13. However, GO annotations do not suggest any relationship between these two proteins; only the broad category of biosynthesis is the common functional annotation between them. Following the same line of thought, URA10 might be interacting with RPS20, GPI13 or MNE1, but GO annotations alone again are insufficient to suggest such possible interactions, and further experimental evidence is necessary.

### Functional modules assemble proteins with similar functions and biological processes

For each eigenvector of the interaction matrix, we have analyzed the similarities of Gene Ontology (GO) annotations within a given cluster using FunSpec[82], a web-based tool. Table 5 shows the results of these assignments for three representative eigenclusters. Most significantly, almost half of the important proteins in each case can be assigned to specific protein functional classes or biological processes. Overall, for these cases, significant proteins are not always restricted to single functions, but rather encompass a spectrum of functions related to cell organization and biogenesis; cell growth and maintenance; and nucleic acid metabolism. We also provide the statistical significances of the functional clustering by including p-values in brackets in Table 5. The low p-values substantiate the high statistical significance of these proteins' assignments, and therefore support our view that functionality is an intrinsic, dominant property within these clusters. However, how can we interpret the functions of the remaining proteins that could not be assigned to these functional classes and biological processes? Our aim here is to identify any similarity in function within these eigenclusters, so a closer look at these clusters is necessary.

**Figure 8:**
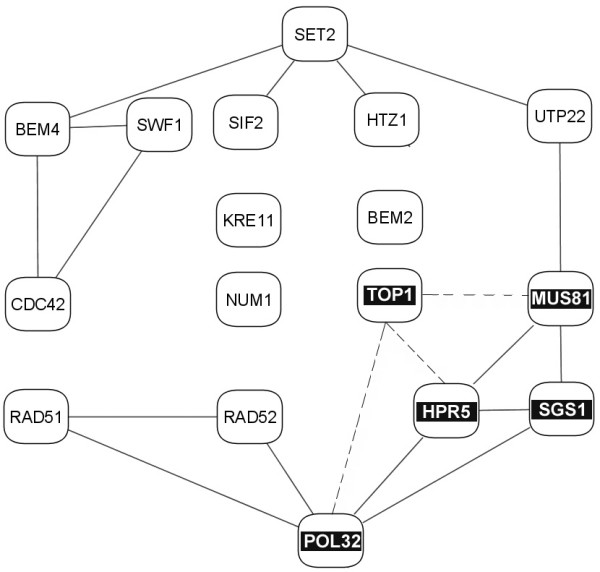
**The sub-network diagram of significant proteins and their interactions for eigenvector #106**. Each node represents a protein, and each edge an experimental interaction in the GRID database. No interactions were indicated for the four central proteins, but by inference their function should be related. Experimental data indicate that SGS1 can be essential[83] in the absence of TOP1, so possibly these two proteins may substitute functionally for one another, thereby suggesting the additional interactions shown by dotted lines.

We show the sub-network diagram for eigenvector #106 in Figure 8, together with the interactions from the protein-protein data. There are 17 significant proteins: 12 of them form a rather linear network and 4 proteins (in the center) remain unconnected. 11 proteins are involved with the cell organization and biogenesis. The remaining 6 proteins are SWF1 (YDR126W), MUS81 (YDR386W), UTP22 (YGR090W), KRE11 (YGR166W), HPR5 (YJL092W), and POL32 (YJR043C). The descriptions of these proteins in Gene Ontology shows that the functions of most of these proteins (except UTP22, which is a "computationally derived uncharacterized ORF") are indeed related to cell cycle or DNA processing, although probably not as statistically significant as for the other 11 proteins: SWF1 is "essential for spore wall formation"; MUS81 is "involved in DNA repair and replication fork stability"; HPR5 is "involved in DNA repair"; and POL32 is related to "DNA synthesis". So, we again see that the proteins in this sub-cluster are connected and functionally related. In addition, the inferred connections with TOP1 (topoisomerase I) yield a cluster of five quite closely related proteins in the lower right hand part of the diagram (shown in reverse color).

Another interesting aspect of this eigencluster shown in Fig. 8 is related to the unconnected proteins: GO annotations show that the unconnected NUM1 (YDR150W), BEM2 (YER155C), and TOP1 (YOL006C) not only take part in cell organization and biogenesis, but also in cell cycle defects. Specifically, NUM1 and TOP1 are the 2 proteins of only 12 proteins assigned to nuclear migration. Therefore, although the presence of interactions is not experimentally verified for these three proteins, they relate closely in function to other proteins in their roles in cell organization. Experimental data indicate that SGS1 can be essential [83] in the absence of TOP1, so possibly these two proteins may substitute functionally for one another, thereby suggesting the additional interactions shown by dotted lines. Further experimentation is needed to test possible interactions of the unconnected KRE11 with other proteins in this eigencluster.

### Functional modules can be utilized to successfully predict new interactions

**Figure 9:**
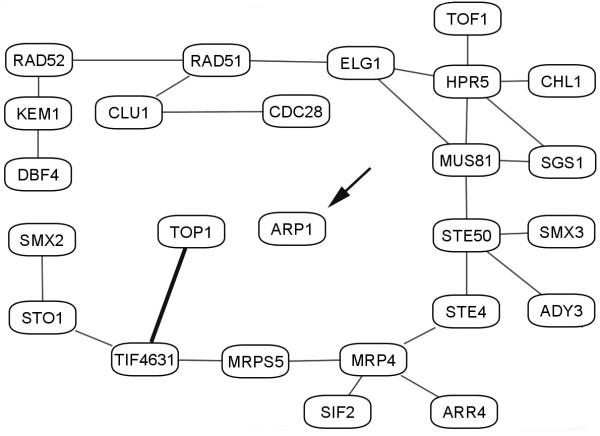
**The cluster corresponding to proteins and interactions in eigenvector #124**. The edges represent interaction information confirmed by experiments. Each node represents a protein. TOP1 and ARP1 are proteins originally found to be unconnected to the network in the cluster in the previous version of the GRID database. The thick line is the newly discovered interaction between proteins TOP1 (YOL006C) and TIF4631 (YGR162W) in the newer version of the GRID database. We expect that protein ARP1 (YHR129C) (shown with an arrow) most likely should also be connected to some other members of the cluster.

After we obtained the preliminary results, new interactions obtained by Krogan *et al*. [59] have been added to the GRID yeast database. Although these additional interactions were not properly curated to ensure non-redundancy, new interaction information proved to be highly useful to test the predictive power of our clustering methodology. As an example, we focus on the 124^th ^eigenvector cluster shown in Fig. 9.

In this eigenvector, all significant proteins except TOP1 and ARP1 are connected to each other forming a full interactive cluster according to older GRID yeast data. According to our functional clustering hypothesis, these two proteins should, however, be connected to the interactive module of other proteins in the cluster. Was this discrepancy due to limitation of our clustering hypothesis or the lack of data? The new interaction data from Krogan *et al*. [59] have confirmed one of our theoretical predictions, since the protein TOP1 (YOL006C) is indeed connected to the interactive module (the new interaction is shown as a heavy line). This is a clear example of the unifying functionality in clusters, supporting our view that the number and the topology of interactions of a given protein are related to its functional role in the cellular processes and that this functionality can be exploited to predict new interactions between proteins found within the same cluster. We also expect the protein ARP1 (YHR129C) in Fig. 9 to be connected to this interaction network, a claim yet to be substantiated by future experiments. There are other cases from the newest data set[59] that confirm the correctness of our prediction methodology (not shown). We are expecting that more of these predictions may be confirmed in the future, since the yeast protein interaction data set is far from being complete. We should note that in a recent paper of Uri Alon's group [84] it has been shown that evolutionarily developed rules of biological regulation are based on error minimization. It is quite possible that functional clusters relate to the error minimization problem, by allowing functionally related proteins to replace each other in multi-regulatory systems.

## Conclusion

We have analyzed the yeast protein interaction network by building a connectivity matrix and by applying singular value decomposition to obtain eigenvectors. We have observed that significant proteins in each eigenvector not only have similar degrees, but also are most likely to interact with each other. These proteins therefore form "functional clusters", and these clusters can guide future experiments to predict new interactions. More detailed interpretations of these networks can be obtained by further studies utilizing information about protein structures. Our method can be especially useful for larger, more complex organisms where collection of the protein interaction data is more complicated. Our results encourage further analyses to confirm that functional clusters detected by our method reflect the modular nature of protein interaction networks and originate from evolutionarily preservation of cellular fitness.

## Methods

### Eigenanalysis of matrices

For a given square matrix **A **of size N × N the eigenvalues λ_i _and eigenvectors **x**_i _(1 ≤ i ≤ N) of size N correspond to the solution of the equation

**Ax **= λ**x **    (1)

The equation **Ax **= λ**x **represents a concise notation of system of linear equations, that have nontrivial solutions only if the determinant

det (**A **- λ**I**_N_) = 0     (2)

where **I**_N _is the identity matrix of size N × N. This is satisfied only for certain values of λ, called eigenvalues, which are roots of the characteristic equation of **A **(that is a polynomial of degree N in λ). For each eigenvalue λ_i _(1 ≤ i ≤ N) there is a corresponding eigenvector **x**_i _that satisfies the equation **Ax**_i _= λ_i_**x**_i_. If some eigenvalues of the matrix **A **are zeros, than the matrix **A **is singular, its determinant det **A **= 0, and generally the inverse matrix **A**^-1 ^that satisfies the relation **AA**^-1 ^= **A**^-1^**A **= **I**_N _does not exist. A standard mathematical approach to deal with such cases is the computation of the matrix pseudoinverse by using singular value decomposition method, which will be discussed in the next sub-section.

### Singular value decomposition

Generally, any matrix **A **of size M × N (with M ≥ N) can be written as a product

***A ***= ***UΛV***^*T *^    (3)

where ***Λ ***is the square matrix of size N × N containing non-negative values λ_1_, λ_2_, ...λ_N _at the diagonal and zeros off-diagonal, and **U **and **V **are matrices of sizes M × N and N × N, respectively, that have orthogonal columns, *i.e*. ∑i=1MUikUin=δkn
 MathType@MTEF@5@5@+=feaafiart1ev1aaatCvAUfKttLearuWrP9MDH5MBPbIqV92AaeXatLxBI9gBaebbnrfifHhDYfgasaacH8akY=wiFfYdH8Gipec8Eeeu0xXdbba9frFj0=OqFfea0dXdd9vqai=hGuQ8kuc9pgc9s8qqaq=dirpe0xb9q8qiLsFr0=vr0=vr0dc8meaabaqaciaacaGaaeqabaqabeGadaaakeaadaaeWbqaaiabdwfavnaaBaaaleaacqWGPbqAcqWGRbWAaeqaaOGaemyvau1aaSbaaSqaaiabdMgaPjabd6gaUbqabaaabaGaemyAaKMaeyypa0JaeGymaedabaGaemyta0eaniabggHiLdGccqGH9aqpiiGacqWF0oazdaWgaaWcbaGaem4AaSMaemOBa4gabeaaaaa@4145@ and ∑i=1NVikVin=δkn
 MathType@MTEF@5@5@+=feaafiart1ev1aaatCvAUfKttLearuWrP9MDH5MBPbIqV92AaeXatLxBI9gBaebbnrfifHhDYfgasaacH8akY=wiFfYdH8Gipec8Eeeu0xXdbba9frFj0=OqFfea0dXdd9vqai=hGuQ8kuc9pgc9s8qqaq=dirpe0xb9q8qiLsFr0=vr0=vr0dc8meaabaqaciaacaGaaeqabaqabeGadaaakeaadaaeWbqaaiabdAfawnaaBaaaleaacqWGPbqAcqWGRbWAaeqaaOGaemOvay1aaSbaaSqaaiabdMgaPjabd6gaUbqabaaabaGaemyAaKMaeyypa0JaeGymaedabaGaemOta4eaniabggHiLdGccqGH9aqpiiGacqWF0oazdaWgaaWcbaGaem4AaSMaemOBa4gabeaaaaa@414B@

It can be shown that the original contact (connectivity) matrix **C **= [C_ij_] for the protein network can be written as

***C ***= ***U***^*T*^***ΛU ***    (4)

where ***Λ ***is the diagonal matrix containing eigenvalues λ_1_, λ_2_, ...λ_N _of ***C***, and ***U ***is the matrix formed from eigenvectors of ***C***. Thus, the elements C_ij _of the contact matrix C can be expressed as

Cij=∑k=1Nλkukiukj     (5)
 MathType@MTEF@5@5@+=feaafiart1ev1aaatCvAUfKttLearuWrP9MDH5MBPbIqV92AaeXatLxBI9gBaebbnrfifHhDYfgasaacH8akY=wiFfYdH8Gipec8Eeeu0xXdbba9frFj0=OqFfea0dXdd9vqai=hGuQ8kuc9pgc9s8qqaq=dirpe0xb9q8qiLsFr0=vr0=vr0dc8meaabaqaciaacaGaaeqabaqabeGadaaakeaacqWGdbWqdaWgaaWcbaGaemyAaKMaemOAaOgabeaakiabg2da9maaqahabaacciGae83UdW2aaSbaaSqaaiabdUgaRbqabaGccqWG1bqDdaWgaaWcbaGaem4AaSMaemyAaKgabeaakiabdwha1naaBaaaleaacqWGRbWAcqWGQbGAaeqaaaqaaiabdUgaRjabg2da9iabigdaXaqaaiabd6eaobqdcqGHris5aOGaaCzcaiaaxMaadaqadiqaaiabiwda1aGaayjkaiaawMcaaaaa@483F@

where *u*_*ki *_denotes the *i*^th ^component of the eigenvector corresponding to the *k*^th ^eigenvalue. Equation 5 can be viewed as the eigenvalue expansion of the contact matrix. From Eq. 5 it follows:

Cii=∑k=1Nλkuki2     (6)
 MathType@MTEF@5@5@+=feaafiart1ev1aaatCvAUfKttLearuWrP9MDH5MBPbIqV92AaeXatLxBI9gBaebbnrfifHhDYfgasaacH8akY=wiFfYdH8Gipec8Eeeu0xXdbba9frFj0=OqFfea0dXdd9vqai=hGuQ8kuc9pgc9s8qqaq=dirpe0xb9q8qiLsFr0=vr0=vr0dc8meaabaqaciaacaGaaeqabaqabeGadaaakeaacqWGdbWqdaWgaaWcbaGaemyAaKMaemyAaKgabeaakiabg2da9maaqahabaacciGae83UdW2aaSbaaSqaaiabdUgaRbqabaGccqWG1bqDdaqhaaWcbaGaem4AaSMaemyAaKgabaGaeGOmaidaaaqaaiabdUgaRjabg2da9iabigdaXaqaaiabd6eaobqdcqGHris5aOGaaCzcaiaaxMaadaqadiqaaiabiAda2aGaayjkaiaawMcaaaaa@44CD@

The eigenvalues with the smallest indices (that correspond to the largest absolute values of λ, as seen in Fig. 1) make the largest contributions, and higher indexed eigenvalues contribute successively less (Eq. 6). We clearly see that the total number of contacts for nodes in the network (especially for those that have the highest connectivities) can be well approximated by a relatively small number of the most dominant eigenvalues, because the majority of eigenvalues shown in Fig. 1 are close to zero and do not provide any significant contributions (Eq. 6).

## Figures and Tables

**Table 1:** The 5 smallest eigenvalues and the proteins related to the corresponding eigenvectors. The number of connections for each protein, and its rank order based on the number of connections are shown in the last two columns.

Eigenvalue index	Eigenvalue	Proteins	# of neighbors	Rank Order
1	-289.02	JSN1 (YJR091C)	288	1
2	-167.12	YKE2 (YLR200W)	166	2
3	-161.21	PAC10 (YGR078C)	160	3
		GIM5 (YML094W)	160	4
4	-161.02	PAC10 (YGR078C)	160	3
		GIM5 (YML094W)	160	4
5	-149.11	YPT6 (YLR262C)	148	5

**Table 2:** The significant proteins in eigencluster #23, their number of connections in the protein-protein interaction network, their corresponding eigenvalues, and GO molecular function annotations.

Proteins	Value	Number of connections	GO Molecular Function annotation
CLA4 (YNL298W)	-0.41	91	protein serine/threonine kinase activity
FKS1 (YLR342W)	-0.34	90	1,3-beta-glucan synthase activity
ARP2 (YDL029W)	-0.09	88	ATP binding; actin binding; structural constituent of cytoskeleton
SMI1 (YGR229C)	-0.05	82	molecular function unknown
PHO85 (YPL031C)	0.06	81	cyclin-dependent protein kinase activity
RVS167 (YDR388W)	0.83	92	cytoskeletal protein binding

**Table 3:** The significant proteins in eigencluster #67, their number of connections in the protein-protein interaction network, their corresponding eigenvalues, and GO molecular function annotations.

Proteins	Value	Number of connections	GO Molecular Function Annotations
MUS81 (YDR386W)	-0.12	45	endonuclease activity
CSM3 (YMR048W)	-0.11	53	molecular function unknown
PSE1 (YMR308C)	-0.07	42	protein carrier activity
CKA1 (YIL035C)	0.05	66	protein kinase CK2 activity
RPC40 (YPR110C)	0.05	67	DNA-directed RNA polymerase activity
HRR25 (YPL204W)	0.08	63	casein kinase activity
GLC7 (YER133W)	0.11	52	protein phosphatase type 1 activity
BUD20 (YLR074C)	0.20	56	molecular function unknown
SEN15 (YMR059W)	0.30	55	tRNA-intron endonuclease activity
HHF1 (YBR009C)	0.87	53	DNA binding

**Table 4:** The significant proteins in eigencluster #4850, their number of connections in the protein-protein interaction network, their corresponding eigenvalues, and GO molecular function annotations.

Proteins	Value	Number of connections	GO Molecular Function Annotations
URA10 (YMR271C)	0.15	2	orotate phosphoribosyltransferase activity
MNE1 (YOR350C)	0.26	3	molecular function unknown
RPS20 (YHL015W)	0.58	2	structural constituent of ribosome
GPI13 (YLL031C)	0.75	1	transferase activity, transferring phosphorus- containing groups

**Table 5:** GO assignments of biological processes and molecular functions for examples of individual eigenclusters with FunSpec.[82] The number of proteins with the same GO annotation is in parenthesis, and the numbers in square brackets are the p-values for the assignments.

Eigenvector	Number of significant proteins	GO Biological Process	GO Molecular Function
106	17	DNA metabolism (10) [3 × 10^-8^], chromosome organization and biogenesis (7) [1 × 10^-7^], nuclear organization and biogenesis (7) [4 × 10^-7^], M phase (7) [3 × 10^-6^], cell organization and biogenesis (11) [4 × 10^-6^]	Double-stranded DNA binding (2) [2 × 10^-4^], single-stranded DNA binding (2) [4 × 10^-4^], DNA helicase (2) [1 × 10^- ^^3^], DNA binding (5) [3 × 10^-3^], Binding (8) [5 × 10^-3^]
267	55	Cell growth and maintenance (50) [9 × 10^-8^], RNA metabolism (14) [2 × 10^-7^], RNA processing (13) [5 × 10^-7^], microtubule-based process (8) [8 × 10^-7^], mRNA processing (9) [9 × 10^-7^], nucleobase, nucleoside, nucleotide, and nucleic acid metabolism (24) [2 × 10^-6^]	Binding (28) [1 × 10^-8^], nucleic acid binding (21) [2 × 10^-7^], RNA binding (13) [2 × 10^-7^], mRNA binding (6) [5 × 10^-5^]
304	51	Nucleobase, nucleoside, nucleotide, and nucleic acid metabolism (36) [1 × 10^-14^], RNA processing (19) [1 × 10^-13^], mRNA processing (14) [4 × 10^-13^], RNA metabolism (19) [1 × 10^-12^], RNA splicing (12) [6 × 10^-11^], mRNA splicing (11) [1 × 10^-10^], metabolism (44) [2 × 10^-10^], cell growth and/or maintenance (49) [7 × 10^-10^]	Binding (32) [8 × 10^-13^], nucleic acid binding (25) [2 × 10^- ^^11^], RNA binding (14) [7 × 10^-9^], mRNA binding (6) [4 × 10^-5^]
